# Changes of and interrelationships between performance-based function and gait and patient-reported function 1 year after total hip arthroplasty

**DOI:** 10.1186/s10195-019-0521-7

**Published:** 2019-03-11

**Authors:** Josefine E. Naili, Margareta Hedström, Eva W. Broström

**Affiliations:** 1 Department of Women’s and Children’s Health, Karolinska Institutet, Karolinska University Hospital, 17176 Stockholm, Sverige; 2 Department of Clinical Science, Intervention and Technology, Karolinska Institutet, Stockholm, Sweden; 3 Department of Orthopedics, Karolinska University Hospital, Stockholm, Sweden

**Keywords:** Performance, Function, Gait, Mobility, Osteoarthritis, Hip, Arthroplasty

## Abstract

**Background:**

The aim of this prospective study is to evaluate the degree of improvement in, and interrelationships between, performance-based function, gait, and patient-reported function 1 year after total hip arthroplasty (THA) in patients with primary hip osteoarthritis (OA).

**Materials and methods:**

Thirty-four patients with hip OA, with a mean age of 67 years (standard deviation, SD 9 years), and 25 age- and gender-matched healthy controls performed three performance-based functional tests, instrumented three-dimensional gait analysis, and completed the Hip disability and Osteoarthritis Outcome Score prior to and 1 year after THA. Effect sizes with 95 % confidence intervals were calculated as measures of the magnitude of improvement in performance after surgery.

**Results:**

Performance-based function displayed large improvements 1 year after THA. Overall gait patterns, quantified using a kinematic and a kinetic gait index, respectively, revealed moderate improvements in kinematics of the operated limb and kinetics of the contralateral limb. Patient-reported function displayed the largest improvement after surgery.

**Conclusions:**

The findings of this study suggest that objectively measured improvements in performance-based function and gait are not in line with patient-reported functional improvements, highlighting the importance of using both subjective and objective methods for evaluating function following THA.

**Level of evidence:**

III.

## Introduction

Total hip arthroplasty (THA) is considered to be a successful surgical approach for patients with osteoarthritis (OA). Traditionally, this surgical procedure was performed on older patients with lower functional demands, therefore functional improvement was not considered to be as important as pain relief. However, an increasing number of younger individuals and those with high functional demands are receiving THA [1, 2]. These individuals desire to maintain an active lifestyle and restore function to a level which allows them to be physically active [3]. Even though research demonstrates decreased pain and improved function, including normalized gait patterns, following THA surgery [4–6], some studies indicate that as many as 10–20 % of patients still have persistent disabilities, limited function, diminished working capacity, gait pattern deviations, and reduced quality of life following THA [7, 8]. Disability associated with THA may be conceptualized as a surgical failure since the indication for surgery is pain and impaired function. Current literature lacks sufficient information about the degree of functional improvement after THA using objective evaluation methods, including gait dynamics, and the interrelationship between outcome measures.

The aim of this study was to evaluate the degree of improvement in performance-based function, kinematic and kinetic overall gait patterns, and patient-reported function 1 year after THA in patients with OA. We further aimed to explore the interrelationships between the methods used to evaluate functional improvement. It was hypothesized that patients with hip OA would display significant improvements in all functional outcome measures at 1 year after THA. However, it was hypothesized that deficits in performance-based function and overall gait patterns would remain as compared with age- and gender-matched healthy controls.

## Materials and methods

A cohort of 40 patients were recruited for this prospective study. Patients with hip OA were recruited from two orthopedic departments in Stockholm, Sweden (Ortho Center Löwenströmska hospital and Karolinska University Hospital) between the years 2011 and 2014. All patients had a diagnosis of unilateral symptomatic primary hip OA and were scheduled for THA within 1 month after baseline evaluation (Table 1). Additional inclusion criteria were ability to walk 10 m repeatedly without use of a walking aid, and ability to understand verbal and written information in Swedish. Exclusion criteria were previous major orthopedic surgery in the lower limbs, severe back pain or other lower-extremity joint pain, rheumatoid arthritis, diabetes mellitus, neurologic disease, and/or other condition affecting walking ability. A control group consisting of 25 healthy individuals without any known musculoskeletal disease or neurological disorder were recruited through a convenience sample of acquaintances between the years 2013 and 2015. The control group was frequency matched to the OA group across five age groups (40–49, 50–59, 60–69, 70–79, and 80–89 years). The regional ethical review board in Stockholm, Sweden approved the study (DNR 2010/1014-31/1). All study participants provided written and verbal informed consent to participate and for the results to be published, in accordance with the Declaration of Helsinki.

**Table 1:** Baseline characteristics of patients with hip osteoarthritis and healthy controls included in the study

	Hip osteoarthritis (*n* = 34)	Healthy controls (*n* = 25)	Difference between groups
Mean (SD) age (years)	66.9 (9.0)	65.7 (9.5)	0.60
40–49 years, *n* (%)	2 (6)	1 (4)	
50–59 years, *n* (%)	7 (21)	5 (29)	
60–69 years, *n* (%)	9 (26)	9 (36)	
70–79 years, *n* (%)	15 (44)	9 (36)	
89–89 years, *n* (%)	1 (3)	1 (4)	
Female, *n* (%)	24 (71)	16 (64)	
Mean (SD) body mass index (kg/m^2^)	26.6 (3.8)	24.9 (2.9)	0.07
Mean (SD) body weight (kg)	76.3 (15)	72.8 (12.2)	0.35
Mean (SD) height (cm)	169 (10)	171 (8)	0.40
Mean (SD) symptom duration (years)	4.3 (3.4)	NA	
Kellgren–Lawrence score (1–4)			
1–2, *n* (%)	–	NA	
3, *n* (%)	10 (29)	NA	
4, *n* (%)	24 (71)	NA	
Use of analgesics			
Daily use, *n* (%)	19 (56)	NA	
If necessary (when needed), *n* (%)	13 (38)	NA	
Never (rarely), *n* (%)	2 (6)	NA	

### Performance-based function

Performance-based function was evaluated using three different tests, including the Five Times Sit to Stand (5STS) test [9], the Timed Up and Go (TUG) test [10], and the Single Limb Mini Squat (SLMS) test [11]. The 5STS test is conducted by recording the time required by the participant to stand up, from a seated position, five times as quickly as possible [9]. The test was performed twice, and the best (lowest) value was used in the analysis. The TUG test is conducted by recording the time it takes the participant to rise from a chair, walk 3 m at a self-selected speed, turn 180°, and return to a seated position. The SLMS test is performed by recording the maximal number of singe-leg mini squats in 30 s. Fingertip support for balance was provided by a frame placed in front of the participant.

### Three-dimensional gait analysis

All gait analyses were conducted by two experienced physiotherapists and performed at the Motion Analysis Laboratory at Karolinska University Hospital, Stockholm, Sweden. Each test session started with a physical examination using a standardized protocol where anthropometric measures were recorded using calibrated scales. All study participants were instructed to walk barefoot along a 10-m-long pathway at self-selected speed. Recordings were performed in two directions (back and forth). Kinematic, kinetic, and spatiotemporal parameters were collected using an eight-camera motion system (©Vicon Motion Systems Ltd., Oxford, UK) and the Plug-In Gait full-body model [12]. Spatiotemporal parameters were normalized (made nondimensional) according to Hof [13].

Overall gait pattern was evaluated using the Gait Deviation Index kinematics (GDI) and kinetics (GDI-kinetic), which allows comparison of an individual’s gait pattern against those of a reference group [14, 15]. Reference subjects (*n* = 59 for GDI, *n* = 56 for GDI-kinetic) were selected based on age from the control database at the Motion Analysis Laboratory. The GDI was calculated from the pelvis and hip kinematics in all three anatomical planes, the knee and ankle in the sagittal plane, and foot progression in the transversal plane [14]. The GDI-kinetic was calculated from the hip, knee, and ankle moments in the frontal and sagittal plane and total joint power in the hip, knee, and ankle [15]. Each limb is considered independently. A GDI or GDI-kinetic score of ≥ 100 represents normal gait pattern, whereas each 10-point decrement below 100 represents one standard deviation from normal gait and indicates a deviating overall gait pattern in kinematics or kinetics, respectively, in one or more joints. Five gait trials, with clean force plate strikes, were analyzed for each participant, at each test session (pre- and postoperative). The GDI and GDI-kinetic were averaged for these trials to obtain one value for each limb, for each index. MATLAB (R2014a) software was used for all gait parameter calculations.

### Patient-reported function and pain

All participants completed the self-administered Hip disability and Osteoarthritis Outcome Score (HOOS) questionnaire which is considered to be reliable for assessing baseline function and hip-related pain, and change over time [16].

### Surgical technique and postoperative regimes

Five senior orthopedic surgeons from two different hospitals performed the surgeries. All included patients with OA received THA with anterolateral approach. Postoperative regimes allowed full weight-bearing together with use of an appropriate walking aid. According to standard practice at each hospital, patients had no postoperative movement restrictions. Postoperative rehabilitation was performed according to standard practice at each hospital and, thereafter, in a primary care setting of the patient’s choice. The standard postoperative rehabilitation lasted for 2 months after surgery.

### Statistical analysis and sample size

Statistical analyses were performed using IBM SPSS Statistics version 23. A *p*-value below 0.05 was considered statistically significant. Normality of data was assessed by Shapiro–Wilk test and Q–Q plots. To assess change in function prior to and 1 year after THA, paired-sample *t* tests were used. To evaluate the magnitude of change in function, effect sizes (Cohen’s *d*) were calculated, and to obtain a precise estimate of change, 95 % confidence intervals (CI) [17]. Since the control group consisted of a functionally symmetric population, we arbitrarily chose the right side in the analysis. To evaluate differences in function between patients with OA at the postoperative follow-up and healthy controls, independent-samples *t* tests were used.

To determine postoperative improvement in performance-based function, the previously proposed minimally detectable change (MDC) for performance on the 5STS test [18] and major clinically important improvement for the TUG test [19] were used as cutoffs for improvement. At the 1-year follow-up, patients with OA were grouped and compared based on change in performance on the 5STS and TUG test according to these thresholds, i.e., reduction in time by ≥ 2.5 s for the 5STS test, and reduction in time by ≥ 1.14 s for the TUG test indicated “improved function,” whereas reduction in time by < 2.5 s (for the 5STS test) and < 1.14 s (for the TUG test) or an increase in time indicated “unchanged function.” To be classified as having improved function, performance on both tests had to be improved beyond the cutoff.

The sample size needed to detect a difference of 2.5 s in performance on the 5STS test and a difference of 5 GDI units, respectively, between patients with hip OA and healthy controls, with the power set at 0.8, was 32 and 24 subjects, respectively, in the hip OA group. Sample size calculations were made based on pilot data.

## Results

**Fig. 1:**
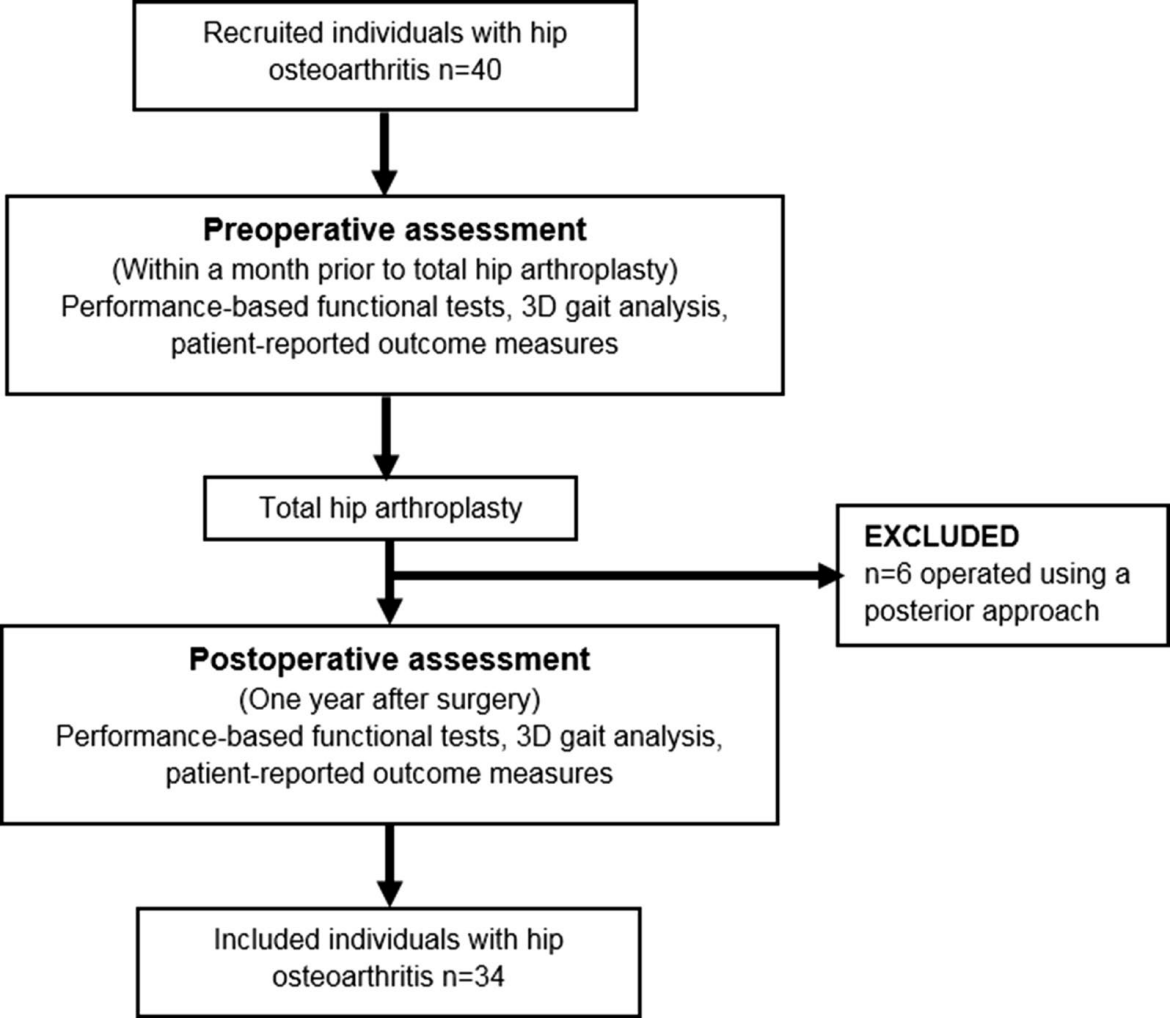
Flowchart of included study participants with hip osteoarthritis, test procedures, excluded individuals, and study participants completing the 1-year follow-up

Out of the 40 patients recruited at baseline, 6 received THA with posterior approach and were thus excluded from the study. Thirty-four patients completed the preoperative assessment and the 1-year follow-up and were included in this study (Fig. 1). The excluded individuals with hip OA did not differ from the studied OA group with regards to age, weight, BMI, or years with symptomatic hip OA.

### Change in performance-based function

Significant improvements were found in all three performance-based tests at 1 year after surgery (Table 2). Change in performance in all three tests displayed large effect sizes, with the largest effect size for the TUG test, a test representing functional mobility and dynamic balance.

**Table 2:** Functional outcomes at baseline prior to total hip arthroplasty and at 1-year follow-up in patients with hip osteoarthritis and healthy controls

	Hip osteoarthritis (*n* = 34)				Healthy controls (*n* = 25)
	Pre-THA mean (SD)	Mean change (SD)	Effect size (95 % CI)	Post-THA mean (SD)	Control group mean (SD)
Performance-based function					
Five Times Sit to Stand Test	15.7 (6.8)	−4.1 (6.7)*	0.9 (0.3–1.4)	11.6 (2.9)	9.9 (2.9)*
Timed Up and Go Test	12.5 (2.2)	−1.6 (2.1)	1.1 (0.5–1.6)	10.9 (1.6)	8.8 (1.4)
Single Limb Mini Squat test	19.6 (7.7)	4.7 (7.9)*	0.8 (0.3–1.4)	24.4 (9.1)	29.3 (10.9)
Overall gait pattern					
GDI, operated limb	85.3 (9.0)	6.9 (13.3)*	0.7 (0.3–1.2)	92.2 (11.2)	96.6 (9)
GDI, nonoperated limb	89.6 (9.7)	4.5 (12.1)*	0.5 (0.0–1.0)	94.1 (9.3)	96.6 (9)
GDI-kinetic, operated limb	92.5 (6.3)	2.8 (9.5)	0.4 (0.0–0.9)	95.4 (8.6)	100 (8.6)*
GDI-kinetic, nonoperated limb	90.4 (8.3)	4.7 (10.1)*	0.7 (0.2–1.2)	95.1 (10.7)	100 (8.6)
Time and distance parameters					
Walking speed (m/s)	1.1 (0.3)	0.1 (0.3)*	0.8 (0.3–1.3)	1.2 (0.2)	1.3 (0.2)*
Normalized walking speed	0.36 (0.05)	0.04 (0.06)	0.9 (0.4–1.5)	0.40 (0.06)	0.44 (0.06)*
Stride length (m)	1.14 (0.13)	0.08 (0.09)	1.2 (0.6–1.7)	1.21 (0.12)	1.34 (0.11)
Patient-reported function					
HOOS symptoms	40 (14)	42 (17)	3.4 (2.5–4.4)	82 (14)	95 (6)
HOOS pain	44 (12)	42 (17)	3.6 (2.6–4.5)	86 (13)	97 (5)
HOOS ADL	50 (14)	37 (18)	2.9 (2.0–3.7)	87 (13)	96 (7)*
HOOS sport and recreation	26 (17)	42 (29)	2.1 (1.4–2.7)	69 (24)	98 (9)
HOOS quality of life	28 (15)	44 (19)	3.2 (2.3–4.1)	72 (17)	95 (8)

*ADL* activities of daily living, *GDI* Gait Deviation Index, *HOOS* Hip disability and Osteoarthritis Outcome Score

* *p* < 0.05,  *p* < 0.001. Paired-sample *t* tests were used to compare pre- and postoperative results within the hip osteoarthritis group and are reported in the column for mean change. Independent-sample *t* tests were used to compare postoperative results of the hip osteoarthritis group with the control group and are reported in the column for control group mean

### Change in overall gait pattern and gait parameters

The overall gait pattern, as evaluated using kinematic and kinetic gait summary measures, displayed moderate improvements in kinematic gait patterns of both the operated and contralateral limb (Table 2). In kinetic gait patterns, significant improvements were found in the contralateral limb. Walking speed and stride length displayed significant improvements at 1 year after surgery (Table 2).

### Change in patient-reported function and pain

Out of all outcome measures evaluated within this study, patient-reported pain and function displayed the largest improvements 1 year after surgery. The HOOS pain and symptoms subscales displayed the largest effect sizes (Table 2).

### Postoperative function compared with healthy controls

At 1-year follow-up, patients with THA displayed deficits in their performance-based function as evaluated by the 5STS test and the TUG test compared with the age-matched control group, while performance on the SLMS test was comparable to the control group. The kinematic overall gait pattern was restored 1 year after surgery, as no difference in GDI scores between the OA and control group remained. The kinetic gait pattern remained significantly worse than in the control group, indicating a gait pattern with larger deviations from normal gait patterns. With regards to patient-reported function, all HOOS subscale scores remained significantly lower compared with the control group at 1 year after surgery.

### Functional improvement represented change beyond the minimal detectable change

**Fig. 2:**
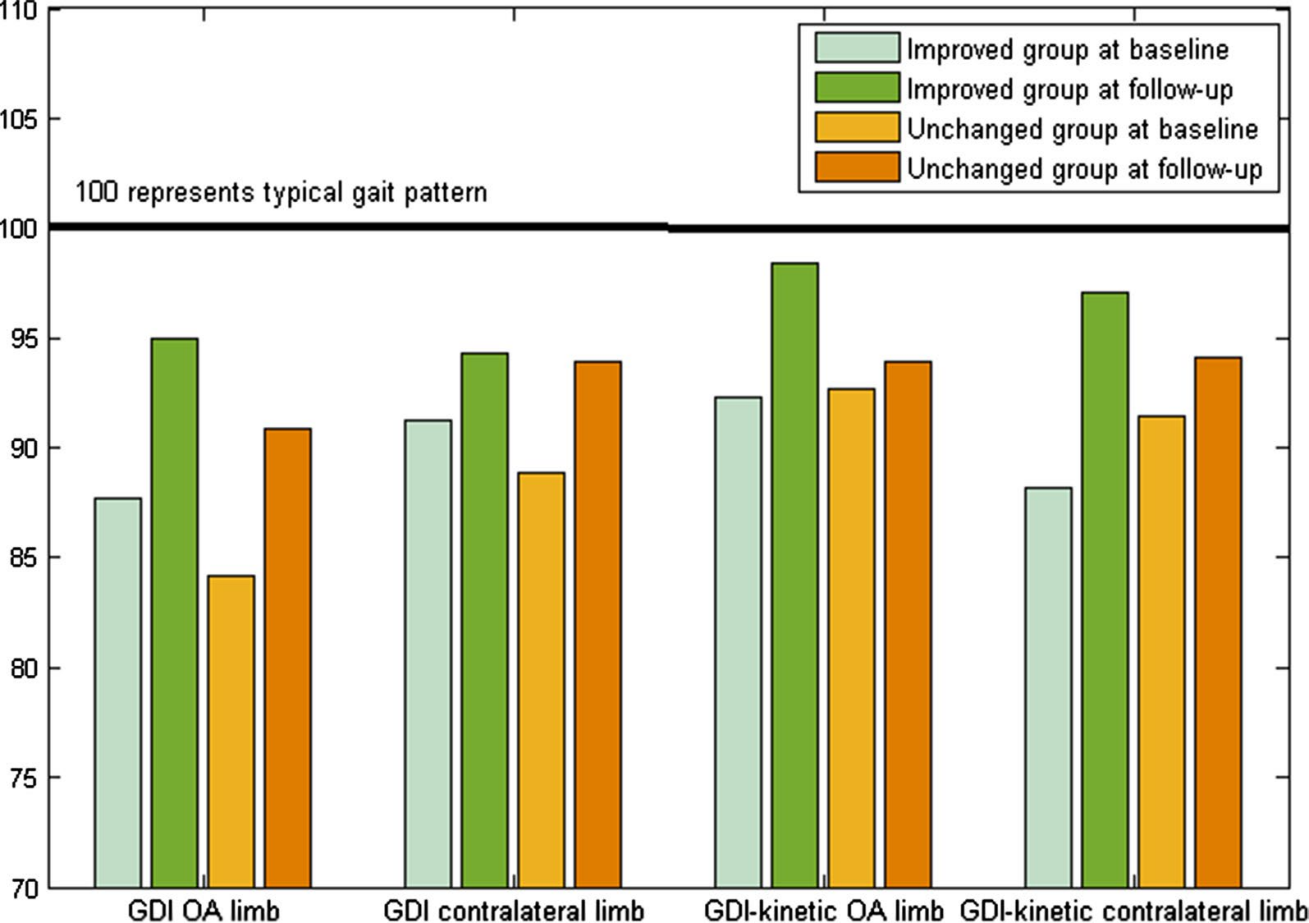
Overall gait pattern, evaluated using the Gait Deviation Index for kinematics (GDI) and kinetics (GDI-kinetic), at baseline and at 1 year after total hip arthroplasty in (*n* = 34) patients with hip osteoarthritis. Patients grouped by postoperative change in performance-based function

**Fig. 3:**
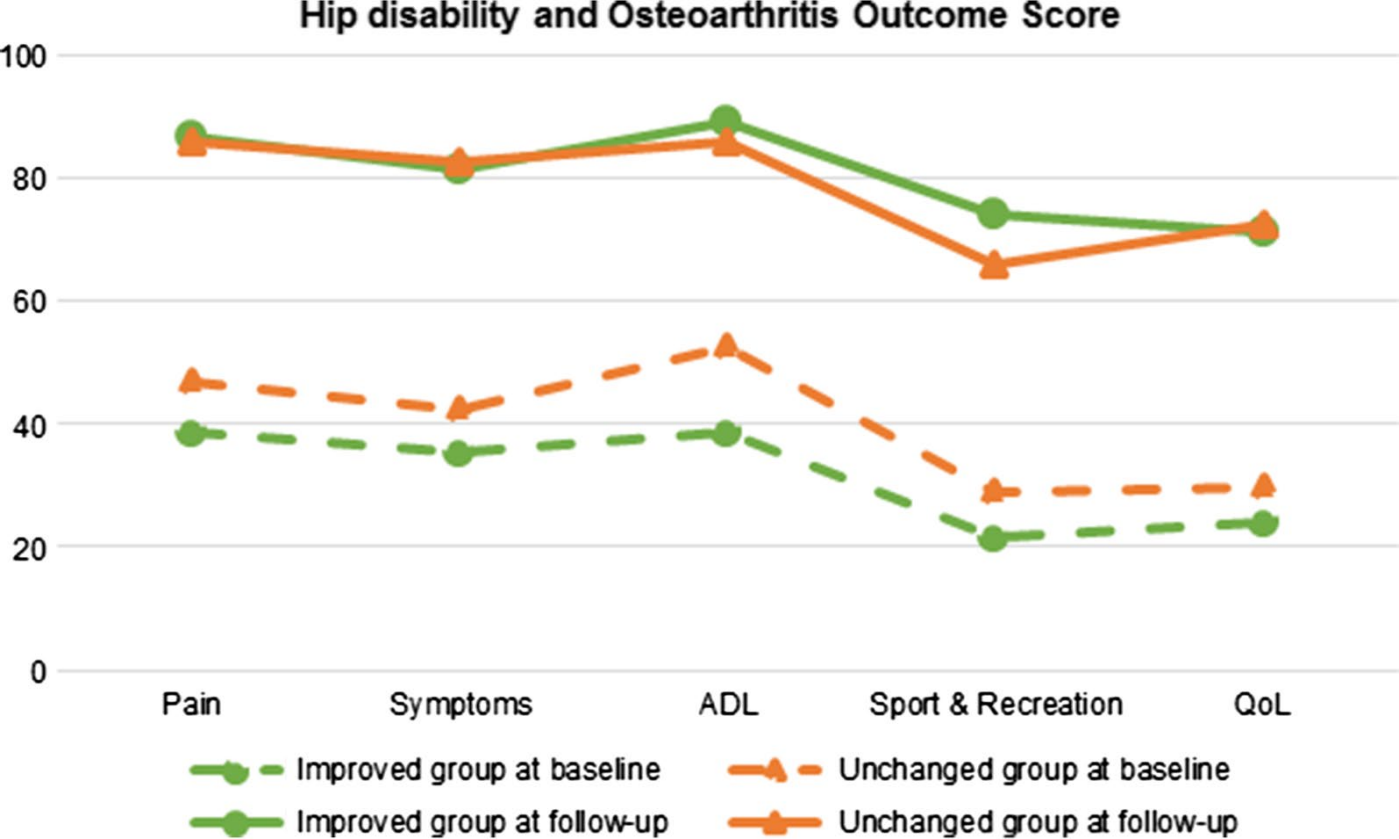
Patient-reported function and pain, evaluated using the Hip disability and Osteoarthritis Outcome Score, at baseline and 1-year follow-up after total hip arthroplasty in (*n* = 34) patients with hip osteoarthritis. Patients grouped by postoperative change in performance-based function. ADL, activities of daily living; QoL, hip-related quality of life

Using the cutoffs of 2.5 s on the 5STS test and 1.4 s on the TUG test to classify performance-based function in patients with THA as “improved” or “unchanged,” 11 (32 %) out of 34 were classified as having improved their performance-based function. Twenty-three (68 %) were classified as having unchanged performance-based function at the follow-up. When comparing change in overall kinematic and kinetic gait patterns between patients improving in performance-based function and those unchanged, no significant differences were found (Fig. 2). When comparing patient-reported function, assessed by HOOS, between patients improving in performance-based function and those unchanged, no significant differences were found (Fig. 3).

## Discussion

This study evaluated changes in function 1 year after THA using an anterolateral approach, and compared postoperative function with an age-matched healthy control group. The findings of the present study display moderate to large improvements in function using three different and established measurement constructs for evaluating function [7, 18–20] following THA surgery. The largest improvements in function, in terms of effect size, were found in patient-reported outcome measures, followed by performance-based function, and lastly kinematic and kinetic gait summary measures.

The largest improvement following surgery was found in the HOOS pain and symptoms subscales, indicating that surgery was successful in reducing pain and hip-related symptoms. The HOOS function in ADL and function in sports and recreation subscales also displayed large effect sizes, indicating that surgery had a huge impact not only on pain and symptoms, but also patient-reported function. Compared with the control group, lower HOOS scores remained in all subscales at the 1-year follow-up.

It was hypothesized that functional measures would improve, although not be restored and comparable to an age-matched healthy control group. Contrary to this hypothesis, no differences remained in kinematic overall gait patterns between patients with THA and the controls at 1 year after surgery. This finding indicates that the overall kinematic gait pattern is as close to a normal gait pattern in patients with a THA 1 year after surgery as in an age-matched healthy control group. Previous studies using the kinematic GDI to evaluate changes in gait patterns have reported deficits compared with healthy controls when gait was evaluated at 6 months after THA surgery [7]. Consequently, the findings of the present study indicate that at least 1 year is needed to restore the kinematic gait pattern following THA surgery.

Only in about one-third of patients with THA did performance-based function improve beyond the previously established threshold for what constitutes a minimal detectable change in performance on the 5STS and TUG tests. Comparisons made between those improving in their performance-based function and those unchanged in how they rated their function and pain, and how their overall gait patterns changed after surgery, revealed no differences between groups. These results could be subjected to type 2 errors with too few individuals in each group. On the other hand, they could also suggest that neither gait summary measures nor patient-report questionnaires measure the same construct as the performance-based test, a finding that supports previous research stating that patient-reported outcomes and performance-based tests are complementary and should be used together [20–22].

This study suffers from a few limitations that should be acknowledged. First, patients with hip OA included in this study were relatively healthy, without comorbidities, and were able to ambulate without use of a walking aid. This limits the generalizability of our findings. Second, the postoperative rehabilitation following THA was not standardized. Patients with THA performed rehabilitation according to standard practice at each hospital and later in a primary care setting of their choice for varying lengths of time. Third, the MDC of the 5STS test and the TUG test used in this study is based on other study samples [18, 19]. Consequently, caution should be taken not to overestimate the importance of these particular thresholds. The strengths of the present study include its prospective study design, use of an age-matched healthy control group for all outcome measures, and use of evaluation methods representing three different measurement constructs. This study provides new information using a comprehensive assessment of function prior to and 1 year following THA using an anterolateral approach.

In conclusion, 1 year following THA all functional outcome measures displayed improvements, with the largest effect size observed for patient-reported function. Performance-based function and overall gait patterns improved after surgery, albeit with a smaller magnitude of change than patient-reported function. These findings suggest that objectively measured improvements in performance-based function and gait are not in line with patient-reported functional improvements, highlighting the importance of using both subjective and objective methods for evaluating function following THA.
